# Draft Genome Sequence of a *Klebsiella pneumoniae* Carbapenemase-Positive Sequence Type 111 *Pseudomonas aeruginosa* Strain

**DOI:** 10.1128/genomeA.01663-15

**Published:** 2016-02-11

**Authors:** Gabrielle A. Dotson, NISC Comparative Sequencing Program, John P. Dekker, Tara N. Palmore, Julia A. Segre, Sean Conlan

**Affiliations:** a National Human Genome Research Institute, Bethesda, Maryland, USA; b NIH Intramural Sequencing Center, Rockville, Maryland, USA; c NIH Clinical Center, Bethesda, Maryland, USA

## Abstract

Here, we report the draft genome sequence of a sequence type 111 *Pseudomonas aeruginosa* strain isolated in 2014 from a patient at the NIH Clinical Center. This *P. aeruginosa* strain exhibits pan-drug resistance and harbors the *bla*KPC-2 gene, encoding the *Klebsiella pneumoniae* carbapenemase enzyme, on a plasmid.

## GENOME ANNOUNCEMENT

*Pseudomonas aeruginosa* is a Gram-negative, opportunistic bacterium that can exhibit high levels of natural resistance, including to antibiotics of last-resort, such as many beta-lactams. *P. aeruginosa* colonizes the lungs, urinary tract, and skin wounds and consequently puts patients with cystic fibrosis, urinary tract infections, and burn injuries, among other conditions, at the greatest risk (1).

*P. aeruginosa* strain AATYA was isolated from a perirectal swab collected upon admission of a patient who had previously received hospital care in Colombia, South America. *P. aeruginosa* AATYA is pan-resistant to antibiotics, including cefepime, ceftazidime, piperacillin-tazobactam, amikacin, tobramycin, gentamicin, levofloxacin, and aztreonam, but is sensitive to colistin. *P. aeruginosa* AATYA is also resistant to meropenem, imipenem, and doripenem.

Genomic DNA and a sequencing library were prepared from *P. aeruginosa* AATYA using the Maxwell 16 nucleic acid purification system (Promega) and the Nextera library kit (Illumina). Sequencing was performed on the Illumina MiSeq instrument. The resulting paired-end reads were assembled using SPAdes version 3.5.1 (2). Pilon, a variant detection and assembly refinement tool, was subsequently applied to ensure a minimal assembly error rate (3). The Pilon-improved SPAdes assembly yielded a total of 135 contigs with an *N*_50_ of 171,911 bp and an average contig size of 52,616 bp. The largest contig was 720,085 bp.

*P. aeruginosa* AATYA has a total size of 7,103,193 bp and an average G+C content of 64%. The NCBI Prokaryotic Genome Annotation Pipeline predicted 6,549 protein-coding genes, and 40 genes are suspected to be plasmid-derived. Also identified were 67 pseudogenes, 57 tRNAs, 6 rRNAs, and 4 non-coding RNAs.

Sequence typing of the genome assembly, places *P. aeruginosa* AATYA in the clinically important ST111 group, considered a high-risk clone (4). There are two published ST111 genomes, a European outbreak strain PA38182 (HG530068) (5) and the Carb 01 63 strain from the Netherlands (CP011317). Those strains, and indeed most carbapenem-resistant ST111 strains, carry the VIM metallo-beta-lactamase, although *Klebsiella pneumoniae* carbapenemase (KPC)^+^ strains have been reported in Colombia (4, 6). *P. aeruginosa* AATYA lacks a VIM gene and carries a *bla*KPC-2 gene in the context of a Tn*4401*b transposon. The Tn*4401*b cassette is contained on a single 33,551 bp contig that is present at 2.9-fold coverage relative to the bacterial chromosome and shares some similarity with other *Pseudomonas* plasmids pCT14 (DQ126685) and pPC9 (CP003739). However, the maximum identity between the AATYA plasmid and the closest reference plasmid, outside the Tn4401a transposon, is 95% and neither pCT14 nor pPC9 carry a *bla*KPC gene. Transferrable resistance genes are a growing concern in *P. aeruginosa* (7), and the genome of *P. aeruginosa* AATYA provides the first genome sequence of an ST111 strain carrying the *bla*KPC gene on a putative plasmid.

### Nucleotide sequence accession numbers.

This whole-genome shotgun project has been deposited at DDBJ/EMBL/GenBank under the accession number LODN00000000. The version described in this paper is the first version, LODN01000000.
